# Successful Closure of Post-pneumonectomy Bronchopleural Fistula With Suture Repair Reinforced With Porcine Acellular Dermal Matrix (Permacol) and Hydrogel Sealant (Progel): A Case Report

**DOI:** 10.7759/cureus.28529

**Published:** 2022-08-29

**Authors:** Adam Djouani, Patrick Hurley, Savvas Lampridis, Andrea Bille

**Affiliations:** 1 Thoracic Surgery, Guy's and St Thomas' NHS Foundation Trust, London, GBR

**Keywords:** pneumonectomy, case report, bronchopleural fistula, air leak sealant, acellular dermal matrix

## Abstract

Bronchopleural fistula (BPF) is a feared and potentially life-threatening complication of pneumonectomy. Clinical features such as a productive cough and subcutaneous emphysema raise suspicion of BPF with CT imaging and bronchoscopy providing a definitive diagnosis. In light of the significant morbidity and mortality associated with the condition, a significant proportion of cases necessitate surgical repair of the bronchial stump.

Currently, there is no consensus on optimal surgical strategy. Traditionally, various vascularised tissue flaps, including pericardial fat pad, omentum, and muscle, have been used to buttress the repaired stump, with varying success rates. In light of this, novel approaches have been devised with the aim of achieving more consistent surgical outcomes. In this case report, we describe a novel approach to reinforcing the suture repair using porcine dermal collagen matrix (Permacol, Medtronic, Minneapolis, MN) and hydrogel sealant (Progel, BD, Franklin Lakes, NJ) to achieve successful closure of a BPF in an adult male patient following pneumonectomy for squamous cell carcinoma.

The use of porcine dermal collagen matrix covered with hydrogel sealant is a viable alternative to traditional BPF closure strategies and can achieve good patient outcomes. This technique has several benefits including cost-effectiveness and sparing of native tissues, and it is technically straightforward. Further studies are required to compare the clinical outcomes of this and other novel techniques with traditional BPF closure approaches.

## Introduction

Bronchopleural fistula (BPF) remains a major complication of pneumonectomy, with an estimated incidence of 2% [1] and mortality ranging from 19.1% to 57.4% [2,3]. Risk factors include smoking, diabetes mellitus, postoperative pulmonary infection, and prolonged mechanical ventilation [4]. Patients may present with dyspnoea, productive cough of serosanguineous fluid or purulent sputum, fever, and subcutaneous emphysema. Chest radiography demonstrates a decreasing fluid level, with CT and bronchoscopy providing a definitive diagnosis. In selected cases, alveolopleural fistulas may be managed conservatively with chest tube drainage or endoscopically. However, for BPF, surgical repair of the bronchial stump remains the treatment of choice [5]. To date, there are no randomised controlled trials demonstrating an optimal surgical strategy. In this article, we report the successful closure of a BPF with a porcine-derived acellular dermal collagen implant (Permacol, Medtronic, Minneapolis, MN) reinforced with a hydrogel sealant (Progel, BD, Franklin Lakes, NJ) in an adult patient.

## Case presentation

A 62-year-old male with a chronic productive cough was found to have a prominent left pulmonary hilum on the chest radiograph. He subsequently underwent a thoracic CT and an [18F]-fluorodeoxyglucose positron emission tomography integrated with CT. The investigations revealed a soft-tissue mass in the left hilum that measured 17 mm in maximal dimension, which had a maximum standardised uptake value of 7.4 and involved the pulmonary artery. A histological diagnosis was not made preoperatively because the lesion raised suspicion for a primary bronchogenic carcinoma on imaging, with a presumptive clinical stage of cT1bN1M0. The patient was a smoker with a 50-pack-year history and had a diagnosis of asthma/chronic obstructive pulmonary disease (COPD) overlap syndrome. No other comorbidities were present. He had an Eastern Cooperative Oncology Group performance status of 1, and pulmonary function test results were as follows: forced expiratory volume in the first second (FEV1) of 62.2%, forced vital capacity (FVC) of 92.9%, FEV1/FVC ratio of 69.2%, and transfer factor of the lung for carbon monoxide of 65.6% of the predicted values. Four weeks after his referral to our department, he underwent a left pneumonectomy and lymph dissection through an open thoracotomy. His operation was uneventful. Postoperatively, he developed non-severe hospital-acquired pneumonia, which was treated empirically with oral antibiotics as cultures were negative. The patient responded well, and inflammatory markers normalised. Serial chest radiographs demonstrated an increasing fluid level in the left thoracic cavity, and he was discharged home on the fifth postoperative day. Histopathological analysis of the resected specimens showed a squamous cell carcinoma of the lung that measured 17 mm in maximum diameter without lymph node involvement (stage pT1bN0M0 according to the 8th edition of the lung cancer stage classification), which was completely resected (R0).

**Figure 1:**
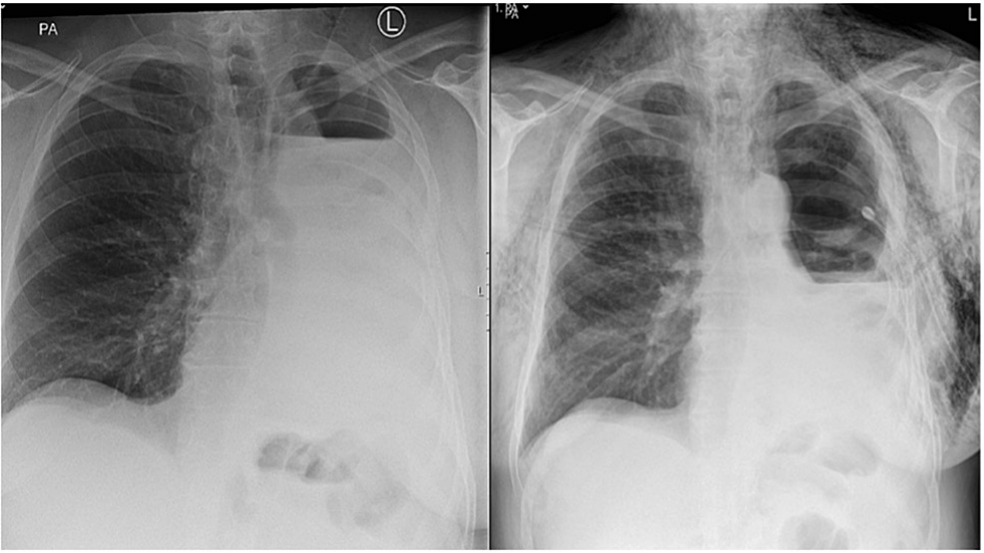
A comparison of the chest radiograph after pneumonectomy prior to hospital discharge (left) with that following clinic review five weeks later (right)

**Figure 2:**
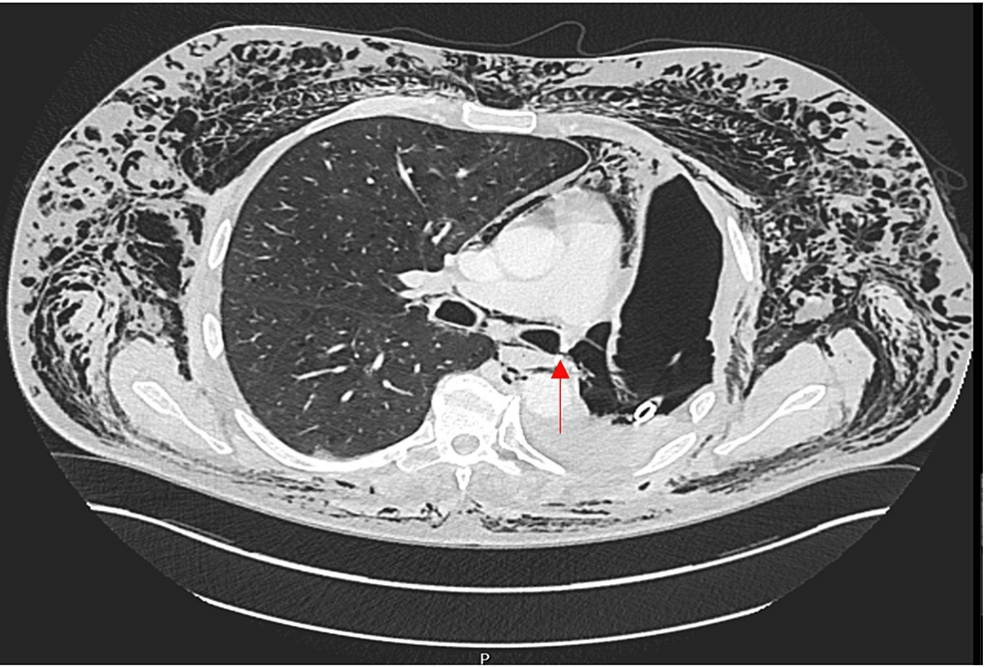
CT scan demonstrating the presence of a bronchopleural fistula in the posterior edge of the left bronchial stump following pneumonectomy (arrow) CT: computed tomography

His first follow-up was in the outpatient clinic three weeks later, when he reported a few days’ history of deteriorating dyspnoea and swelling in his precordium and neck with accompanying dysphonia. A chest radiograph (Figure 1) demonstrated widespread subcutaneous emphysema and a drop in the fluid level in his left hemithorax compared to previous imaging. A BPF was highly suspected, and hence a chest tube was inserted to drain the chest cavity. A subsequent chest CT showed a residual loculated collection and a fistula in the posterior edge of the left bronchial stump (Figure 2). Two days later, he underwent flexible bronchoscopy, which confirmed a BPF in the posterolateral corner of the left pneumonectomy stump.

**Figure 3:**
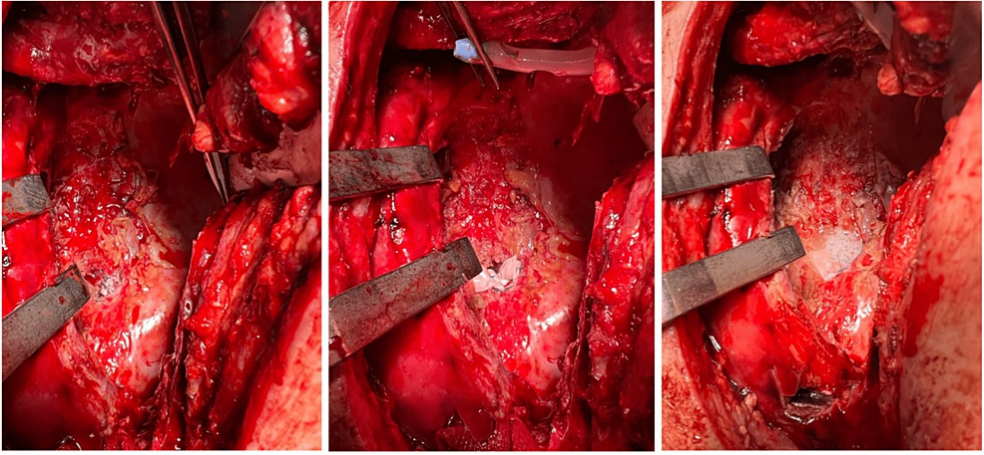
Intraoperative images demonstrating closure of BPF with porcine-derived acellular dermal collagen implant* *Permacol, Medtronic, Minneapolis, MN BPF: bronchopleural fistula

A decision was made to proceed with a redo left thoracotomy and closure of the BPF six days after his bronchoscopy, allowing time for the patient to be optimised with antibiotic treatment, a high protein diet, and intensive chest physiotherapy. The left main bronchial stump was debrided and directly repaired with 3/0 and 4/0 interrupted polypropylene sutures (Prolene, Ethicon, Bridgewater, NJ). The bronchial repair was then reinforced with Permacol, which was fixed with interrupted 4/0 Prolene sutures and polytetrafluoroethylene pledgets (Figure 3). Progel was subsequently applied on top of the repair. The repaired stump was checked underwater with airway pressures up to 35 cmH_2_O, without evidence of an air leak. The thoracic cavity was washed with povidone-iodine and gentamicin. Two chest drains were inserted at the base and apex of the thoracic cavity, respectively, and the thoracotomy was closed in a standard fashion.

Postoperatively, the patient developed a wound seroma that was aspirated and decompressed. The chest drains were removed on the third and fourth postoperative days, respectively. Serial chest radiographs confirmed a rising fluid level in the left hemithorax and resolving subcutaneous emphysema. He required prolonged intravenous antibiotic therapy, as pleural debris sent for cultures intraoperatively grew Streptococcus viridans, and he was discharged from the hospital three weeks after his surgery. He was reviewed in the outpatient clinic four weeks following discharge, and he reported that he had been going for regular walks and building his fitness. A repeat chest radiograph confirmed resolved subcutaneous emphysema and an increasing fluid level.

## Discussion

BPF is a life-threatening complication that can occur after pneumonectomy. Risk factors include advanced age, male sex, right-sided surgery, poorly controlled diabetes mellitus, and radiation therapy [6]. The clinical presentation occurs in acute, subacute, or chronic forms. Subacute and chronic BPF present with a more insidious course and varying levels of respiratory compromise. Typical symptoms include expectoration of purulent sputum, dyspnoea, subcutaneous emphysema, and mediastinal shift [7]. Patients may also develop aspiration pneumonia or empyema, which carries significant morbidity and mortality [8]. Diagnosis is often suspected based on serial chest radiographs that demonstrate a decreasing fluid level in the post-pneumonectomy hemithorax. CT combined with bronchoscopy is used to confirm the presence of a BPF. Several surgical techniques have been employed to repair BPF. Traditionally, repair has involved direct suture closure and the use of pericardial or thoracic muscle flaps [9]. While effective in certain cases, the results remain variable, with studies reporting a success rate of up to 59% and a mortality rate of up to 56% [10]. In light of this, novel approaches, such as the use of a mesenchymal stem cell-seeded matrix, have been recently devised [11]. However, such techniques require specialised equipment and are costly.

Permacol is a porcine acellular dermal collagen matrix that has been successfully used in several surgical specialties for applications that include chest wall reconstruction and abdominal wall hernia repair [12,13]. Permacol is cost-effective and has been shown to result in savings of approximately 80% compared to similar alternatives [14]. The acellular nature of Permacol means that it is immunologically inert, allowing it to be integrated into host tissues without any allergenic or inflammatory response. Furthermore, its crosslinked structure gives Permacol a high tensile strength and makes it resistant to collagen-degrading enzymes, making it ideal for use in tissues of the respiratory system [15,16]. Despite these advantages, the use of Permacol within the thoracic cavity is very limited to date. Ruttenstock et al. reported a case of successful BPF closure in an infant using a bronchoscopic approach with Permacol and a fibrin glue plug [17]. In addition, Gooch et al. have demonstrated the successful repair of diaphragmatic hernia with Permacol [18]. To the best of our knowledge, there are no prior cases of BPF repair with Permacol in adult patients reported in the literature.

Progel combines polyethylene glycol and human serum albumin to form a hydrogel sealant that is malleable, highly adherent, and tear-resistant. In a study by Ibrahim et al. involving 11 patients who suffered bronchial fractures during lung resection leading to a high risk of air leak and BPF, the application of Progel was shown to be 100% effective in preventing air leak, while no complications were identified [19]. In addition, in a study involving 2,670 patients, Mortman et al. demonstrated that the use of Progel to seal air leak intraoperatively led to reduced length of stay when compared to other sealants (9.9 vs. 11.3 days; p<0.001) [20]. The rationale behind the use of a combination of Permacol and Progel in addition to suture repair was to increase the likelihood of the successful closure of the fistula. While the use of either biomaterial requires reinforcement with suture repair, it remains to be elucidated whether the use of Permcol or Progel in isolation would provide successful fistula closure and further studies are needed to evaluate this.

## Conclusions

This case report demonstrates that the use of Permacol combined with Progel provides a feasible, safe, and effective approach for the surgical repair of BPF after pneumonectomy. While the use of other techniques, such as vascularised muscle flaps, is currently more prevalent, we feel that this technique is a viable alternative with the benefit of sparing native tissues. It also does not require the resources and technical expertise compared to other novel methods such as the use of mesenchymal stem cells. Further studies are required to compare the outcomes between this technique and other approaches in order to identify the optimal BPF closure strategy.
